# Leaf anatomical traits shape lettuce physiological response to vapor pressure deficit and light intensity

**DOI:** 10.1007/s00425-025-04774-2

**Published:** 2025-07-13

**Authors:** Chiara Amitrano, Murat Kacira, Carmen Arena, Stefania De Pascale, Veronica De Micco

**Affiliations:** 1 Department of Agricultural Sciences, University of Naples Federico II, Portici, Naples, Italy; 2 Department of Biosystems Engineering, University of Arizona, Tucson, AZ USA; 3 Department of Biology, University of Naples Federico II, Naples, Italy

**Keywords:** Controlled environment agriculture, Leaf anatomy, Light–VPD interaction, Phenotypic plasticity, Photosynthesis, Stomatal density, Vapor pressure deficit, Vein density, Vertical farming

## Abstract

**Main conclusion:**

Anatomical plasticity in stomatal, vascular, and mesophyll traits enables lettuce to partially buffer high evaporative and irradiance stress, advancing understanding of crop acclimation under a changing environment.

**Abstract:**

Phenotypic plasticity in leaf anatomical and physiological traits is fundamental for plant acclimation to variable environmental conditions. While the individual effects of light intensity and vapor pressure deficit (VPD) on plant performance are relatively well understood, their interactive influence on leaf structure and function remains underexplored. Here, we investigated the response of *Lactuca sativa* L. var. *capitata* (‘Salanova’) to combinations of two VPD levels (0.78 and 1.4 kPa) and three daily light integrals (DLIs; 8.6, 12.9, and 15.5 mol m⁻^2^ d⁻^1^) in a vertical farming system. Contrary to our initial hypothesis that high irradiance combined with elevated VPD would impair mesophyll development and photosynthetic performance, plants under high VPD and high DLI exhibited pronounced anatomical plasticity. These included a 40% increase in stomatal density, a 24% increase in minor vein density, enhanced palisade mesophyll thickening, and elevated chloroplast surface exposure to intercellular airspaces (Sc), enabling partial maintenance of CO₂ diffusion despite reductions in mesophyll gas phase conductance (g_ias_). Multivariate analyses revealed a strong coordination among anatomical and physiological traits under high VPD, with vascular and stomatal traits emerging as critical nodes. Although plants under low VPD consistently achieved higher biomass, photosynthesis, and water use efficiency, those under high VPD and high light conditions activated structural and biochemical compensations (e.g., increased V*cmax* and J*max*), mitigating the detrimental effects of environmental stress. Our findings emphasize the essential role of leaf anatomical plasticity in facilitating plant acclimation to combined high evaporative demand and irradiance, offering novel insights for optimizing crop performance in controlled environment agriculture.

**Supplementary Information:**

The online version contains supplementary material available at 10.1007/s00425-025-04774-2.

## Introduction

Light intensity and air humidity are two critical environmental drivers influencing plant growth and physiological function. Variations in light intensity affect energy capture, pigment–protein complex assembly, and electron transport within the photosynthetic apparatus (Hikosaka et al. 1999; Park et al. 2019; Croce and van Amerongen 2020; Morales and Kaiser 2020). Even modest changes in irradiance can elicit pronounced alterations in leaf morphology and anatomy, including lamina thickening, changes in mesophyll organization, and redistribution of chloroplasts, which collectively modulate mesophyll conductance and photosynthetic capacity (Poorter et al. 2019; Evans 2021; Flexas et al. 2021). These structural changes are particularly important in species with high plasticity, enabling them to adjust their photosynthetic machinery to fluctuating light conditions or other concurrent environmental stressors (Oguchi et al. 2003). Such plasticity has been recently highlighted in lettuce under variable daily light integrals (Pennisi et al. 2019), indicating that even subtle irradiance variations can significantly alter stomatal and mesophyll traits.

Similarly, vapor pressure deficit (VPD), a measure of atmospheric dryness, strongly influences gas exchange and water loss. High VPD typically reduces stomatal conductance and photosynthesis, impairing biomass accumulation, particularly critical in crop species (Amitrano et al. 2019; Du et al. 2020). In non-crop species, high VPD can similarly impair carbon assimilation, compromising plant survival and the whole ecosystem stability under current climate change scenarios, where drought and atmospheric dryness are intensifying (Grossiord et al. 2020).

While numerous studies have addressed the individual effects of light and VPD in crops, their combined impact and especially the underlying anatomical responses, with consequent eco-physiological performance, remain less explored (Evans 2021; Amitrano et al. 2022a). Emerging evidence suggests that exposure to simultaneous stresses triggers integrated anatomical and biochemical adjustments, as shown by Li et al. (2021) in tomato, emphasizing that multi-stress environments necessitate coordinated structural responses. Understanding how these factors interact to influence leaf anatomical development is crucial for optimizing crop performance under fluctuating environmental conditions.

The development of anatomical traits is often coordinated to optimize structural efficiency. In particular, stomatal density, mesophyll organization, and vein architecture not only influence gas diffusion and water transport, maintaining the balance between water use, carbon assimilation, and transpiration across varying environments, but also constrain the plasticity of physiological traits (Terashima et al. 2011; Blonder et al. 2015). For instance, it is increasingly evident that both stomatal and mesophyll conductance are anatomically regulated (Vialet-Chabrand et al. 2017; Sharkey 2020; Xiong et al. 2023) and that a large fraction of hydraulic resistance resides within the leaf mesophyll (Cochard et al. 2004; Earles et al. 2018). Additionally, some anatomical traits, particularly stomatal density, may be modulated not only through developmental regulation, but also as a passive outcome of differential leaf expansion. For instance, increased lamina expansion under low irradiance may lead to greater spacing between epidermal cells, effectively reducing their areal densities even if their absolute numbers remain unchanged (Carins Murphy et al. 2012). This distinction is important when interpreting density-based responses to light and VPD.

Despite the centrality of these traits, crop research has often prioritized yield-related parameters, overlooking the anatomical basis of stress responses. Recent efforts have proposed the integration of high-throughput phenotyping of anatomical traits to characterize these functional traits, collectively referred to as “anatomics”, which offer a mechanistic understanding of plant acclimation (Strock et al. 2022). In a previous study (Amitrano et al. 2022b), it was pointed out that integrating image-based high-throughput phenotyping with detailed anatomical analysis provides a comprehensive understanding of lettuce responses to changes in VPD and water availability. Notably, high-throughput phenotyping (RGB, IR, chlorophyll fluorescence) failed to detect key structural adjustments—such as changes in mesophyll organization, stomatal and vein density—that were critical for interpreting the plants’ acclimation strategies, underscoring the essential role of leaf anatomy in modulating eco-physiological responses.

Expanding upon these findings, the present study investigates the interplay between VPD and irradiance in shaping the anatomical and physiological plasticity of red-leaf lettuce (*Lactuca sativa* L. var. *capitata* ‘Salanova’) grown in controlled conditions. Plants were exposed to two VPD regimes (0.78 and 1.4 kPa) in combination with three light levels expressed as daily light integrals (DLI): 8.6 (low light), 12.9 (medium light), and 15.5 (high light) mol m⁻^2^ day⁻^1^.

We hypothesized that higher irradiance would amplify the effects of elevated VPD, constraining mesophyll development and limiting gas exchange, and that structural plasticity would mediate the plant’s acclimation capacity. Understanding the interplay between anatomical and physiological responses under combined environmental stresses is critical not only for advancing basic plant science, but also for developing sustainable strategies in controlled-environment agriculture.

## Materials and methods

### Experimental design and plant material

**Fig. 1:**
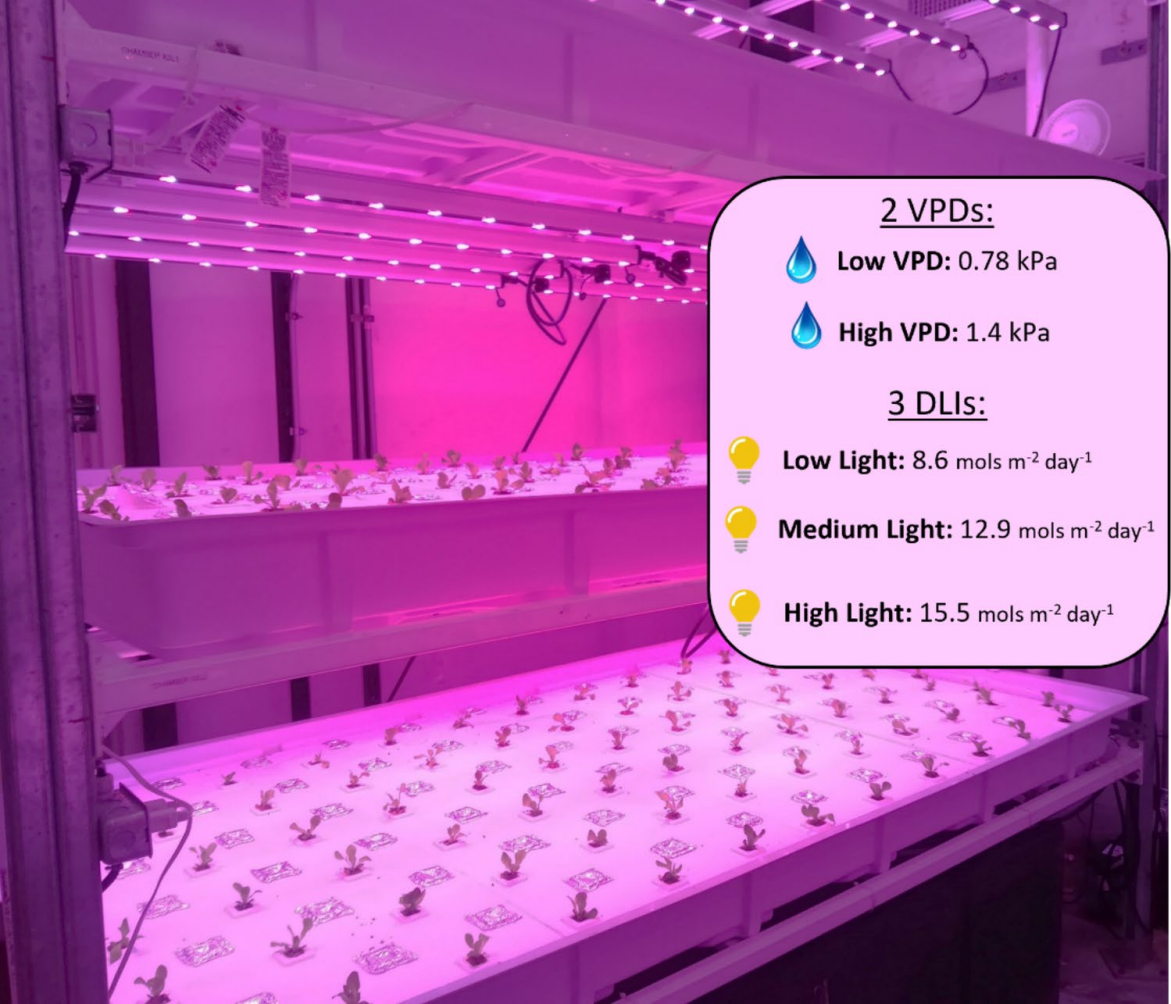
Summary of the experimental design showing one of the vertical farm racks with growing lettuce plants. All the information about the treatments: VPDs and DLIs are reported in the figure

The experiment was conducted in a multi-layer vertical farming research facility at the Controlled Environment Agriculture Center of the University of Arizona (UA-CEAC). Seeds of red ‘Salanova’ lettuce (*Lactuca sativa* L. var. *capitata*) were purchased from Johnny’s Selected Seeds (www.Jonnyseeds.com, Albion, ME, USA) and pre-germinated in rockwool cubes, employing an ebb and flow hydroponic system, in a greenhouse under ambient conditions. Fourteen days after sowing (DAS), uniform seedlings were transplanted into the vertical farm and cultivated for 23 days into a rack containing three levels of deep-water culture hydroponic growing beds (Fig. 1).

Plants were grown in two subsequent cycles, each characterized by a different air VPD: the first cycle was optimal or moderate/low VPD (0.78 kPa) and the second cycle sub-optimal/high VPD (1.4 kPa) while all other environmental and agronomic conditions (light regime, temperature, nutrient supply, seed lot) were kept identical in the two cycles. For simplicity and consistency with figure and table labels, we retained the terms ‘low’ and ‘high’ VPD to refer to the two treatments, although 0.78 kPa is more accurately considered a moderate or optimal value and 1.5 kPa sub-optimal.

During both cycles, each of the three layers contained 78 plants and was exposed to a different daily light integral (DLI): (i) 8.6 mol m^−2^ day^−1^ corresponding to 200 μmol photons m^−2^ s^−1^ (low light, LL); (ii) 12.9 mol m^−2^ day^−1^, corresponding to 300 μmol photons m^−2^ s^−1^ (medium light, ML); and (iii) 15.5 mol m^−2^ day^−1^, corresponding to 360 μmol photons m^−2^ s^−1^ (high light, HL) (Fig. 1). These labels (LL, ML, HL) are used in a relative sense within the experimental context to distinguish treatments that induced distinct anatomical and physiological responses, although none of the light levels reached photosynthetic saturation, as shown in Fig. S3.

Lighting was provided by an LED system (Eclipse F3 LED bar, Illumitex, Austin, TX, USA), positioned 0.4 m above the canopy. The spectral composition was 80% red, 15% blue, and 5% green, with a 12-h photoperiod (8 AM to 8 PM). VPD levels were achieved by means of a de-humidifier (90L/D Parkoo; Guangdong, China). Environmental parameters were logged every 15 min, including air temperature, relative humidity, CO₂ concentration, and PAR; root-zone conditions included electrical conductivity (EC), pH, and dissolved oxygen (DO) (Fig. S1). During the moderate/low VPD cycle (0.78 kPa), the average air temperature was 23.1 ± 0.03 °C and the relative humidity 74.65 ± 0.56%; during the high VPD cycle (1.4 kPa), the average temperature was 23.2 ± 0.3 °C and relative humidity 48.32 ± 0.6%. Further technical details regarding facility setup are reported in Caplan (2018).

### Growth measurements and leaf functional traits

At the beginning of both cultivation cycles, five plants were harvested to assess initial growth parameters: fresh weight (FW), dry weight (DW), number of leaves, and plant area (top-view projected area in cm^2^). FW was measured by harvesting the whole shoot at the substrate level. DW was determined after drying the tissues at 60 °C for at least 3 days until a constant weight was reached. Plant area was quantified using digital image analysis in ImageJ (NIH, Bethesda, MD, USA). These data defined the starting point of the growth curve (1 day after transplanting, DAT) and were used to ensure uniformity between cycles. Sampling continued at 5, 10, 17, and 23 DAT to monitor growth progression. Plants were randomly harvested at each time point, excluding border individuals. Removed plants were not replaced, but gaps were immediately covered to prevent changes in the local microenvironment. Plant spacing ensured no overlap or shading, even at full canopy expansion.

At 23 DAT, six plants per treatment were used to assess leaf functional traits following Cornelissen et al. (2003). Firstly, leaves were scanned to calculate leaf size (leaf lamina area in mm^2^) using ImageJ. Then, FW of each leaf was recorded, and the leaf petiole was submerged in distilled water in the dark for 48 h and then the leaf was reweighted to calculate the saturation weight (SW), whereas DW was obtained by oven drying leaves at 60 °C for at least 3 days, until a constant weight. These parameters were used to evaluate the leaf mass per area (LMA), a proxy for sclerophylly (Witkowski and Lamont 1991), calculated as the ratio between DW and leaf size (g mm^−2^) and the relative water content (RWC %) calculated as the percentage of (FW−DW)/(SW−DW).

### Gas exchange measurements and curves model fitting

Gas exchange measurements were carried out at the end of the growth period on fully expanded leaves, using a portable infrared gas analyzer LI-6400 (LI-COR Biosciences, Lincoln, NE, USA). Parameters measured included CO_2_ net assimilation rate (A_N_, µmol CO_2_ m^−2^ s^−1^), stomatal conductance (g_s_, mol H_2_O m^−2^ s^−1^), transpiration rate (E, mmol H_2_O m^−2^ s^−1^), and instantaneous water use efficiency (iWUE, calculated as the ratio A_N_/E).

Light response curves (A_N_/I) were obtained with the same instrument, exposing the leaves to a photosynthetic photon flux density (PPFD) ranging from 2000 to 0 μmol (photons) m^−2^ s^−1^ (2000, 1500, 1200, 1000, 500, 200, 100, 50 and 0) and waiting at least 15 min for each light intensity to reach a steady state. Temperature, CO_2_ concentration, and VPD inside the leaf chamber were precisely controlled. Curves were fitted using the rectangular hyperbola model by Lobo et al. (2013) to derive useful photosynthetic parameters, such as the maximum assimilation rate (A*max*) and the light compensation point (*Icomp*).

CO_2_ response curves (A_N_/Ci) were obtained on the same plants, by exposing each leaf to eight different air CO_2_ concentrations ranging from 50 to 1000 ppm at a light intensity of 1,200 μmol m^−2^ s^−1^ (considered the saturation irradiance for the species based on A_N_/I curves). To do so, leaves were initially exposed to 400 μmol mol CO_2_^−1^, then the CO_2_ concentration was decreased to 50 ppm and increased again up to 1000 ppm following the *A*_*N*_*-Ci Curve2* auto program, where ten levels of reference CO_2_ (400, 300, 200, 100, 50, 400, 400, 600, 800, and 1000 ppm) were used. Readings were taken after at least 15 min at each step. Curves were fitted following the model by Sharkey et al. (2007). The photosynthetic parameters calculated from the curves were the maximum rate of Rubisco carboxylation (V*cmax*) and the maximum rate of electron transport for the given light intensity (J*max*); both models explain the underlying biochemical limitation of photosynthesis and relate the photosynthetic biochemistry foreseeing the primary production based on prevailing environmental conditions (Von Cammerer 2013).

### Anatomical analyses

Two days before harvest, a healthy fully expanded leaf was sampled from ten plants per treatment, collected in the FAA fixative solution (formaldehyde, glacial acetic acid, 50% ethanol at 5:5:90, by vol.), and stored at 4 °C. Samples were transferred to the Plant and Wood Traits laboratory at the Department of Agricultural Sciences of the University of Naples Federico II to assess possible differences in leaf anatomical traits among treatments. Leaves were dissected into three small segments for the quantification of anatomical traits.

For the characterization of leaf lamina and chloroplast traits, 5 × 5 mm portions were dissected from the central region of the first leaf segment including the midrib, dehydrated in an ethanol series (from 50 to 100%), infiltrated, and embedded in JB4 acrylic resin (Polysciences, Warrington, PA, USA). Semi-thin cross Sects. (5 μm thick) were cut using a rotary microtome, placed on a glass slide, stained with 0.025% toluidine blue O, and mounted with distilled water. Slides were observed under a BX51 light microscope (Olympus); digital images were collected through the Olympus EP50 digital camera at various magnifications and analyzed using the Olympus CellSens 3.2 software to quantify epidermal, palisade, spongy, and total mesophyll thickness, and percentage of intercellular spaces (μm). The percentage of intercellular spaces was also measured, as well as the anatomical mesophyll gas phase conductance (g_ias_, S m⁻^2^) following Syvertsen et al. (1995).

To quantify chloroplasts traits, from pictures taken using a 50 × objective (total magnification: 500 × with 10 × eyepiece), the surface area of the mesophyll cells facing the intercellular space per unit leaf area (Smes) was calculated together with the area of the chloroplast surfaces facing the intercellular space per unit leaf area (Sc) and the ratio Sc/Smes, which represents the fraction of mesophyll cell surface covered by chloroplasts, was then obtained. To convert the length of the cross section to the surface area, a curvature correction factor (F) was determined by assuming that the shape of the palisade tissue cells was a cylinder with flat ends and that the shape of the spongy cells was a spheroid, following Oguchi et al. (2003).

Concerning stomatal traits, leaf abaxial peels were taken centrally in each part of the leaf lamina (avoiding the midrib and margins). For each leaf, measurements were averaged from five microscopy fields (field area of 0.033 mm^2^) obtained from three different peels. Stomatal density was measured as the number of stomata per mm^2^ of the leaf area using ImageJ. Stomatal length was determined considering the length (µm) of the five largest stomata guard cells per ten fields.

To determine vein traits, leaf lamina was chemically cleared following Berlyn and Miksche (1976). To improve the contrast and highlight even the smallest veins, the cleared leaves were stained with safranin and fast green. Each leaf was imaged in five fields of view (field area of 0.15 mm^2^). From those pictures, the vein density (vein length per unit area) was calculated using image J software as the sum of vein densities for 4° veins and higher (mm mm^−2^). The free endings vein per area (FEV) was calculated as the ratio of the free vein endings/leaf area and expressed in n mm^−2^ (Sack and Scoffoni 2013).

### Data analysis

The influence of VPD and DLI on all measured traits was analyzed via two-way ANOVA, followed by Tukey’s HSD post -hoc test (*P* ≤ 0.05), using R software (version 4.4.2).

Photosynthetic curves were evaluated based on R^2^ values and residual sums of squares (SSE). Principal component analysis (PCA) was performed on scaled trait data using the *prcomp()* function in R to identify major axes of variation among treatments. Trait correlation networks were constructed based on Spearman correlations (*P* ≤ 0.05) and visualized using the *igraph* and *ggraph* R packages, with edge thickness proportional to correlation strength. In each network, nodes represent individual traits, while edges correspond to significant correlations between trait pairs. We quantified the following network metrics. (i) Average degree: the average number of connections per node that reflects the extent to which traits co-vary. (ii) Network density: the ratio of observed edges to the total number of possible edges, with higher values indicating a more interconnected network. (iii) Clustering coefficient: the tendency of a trait’s neighbors to be also connected among themselves, which represents local trait coordination or modularity.

## Results

### Growth dynamics under varying VPD and light conditions

**Fig. 2:**
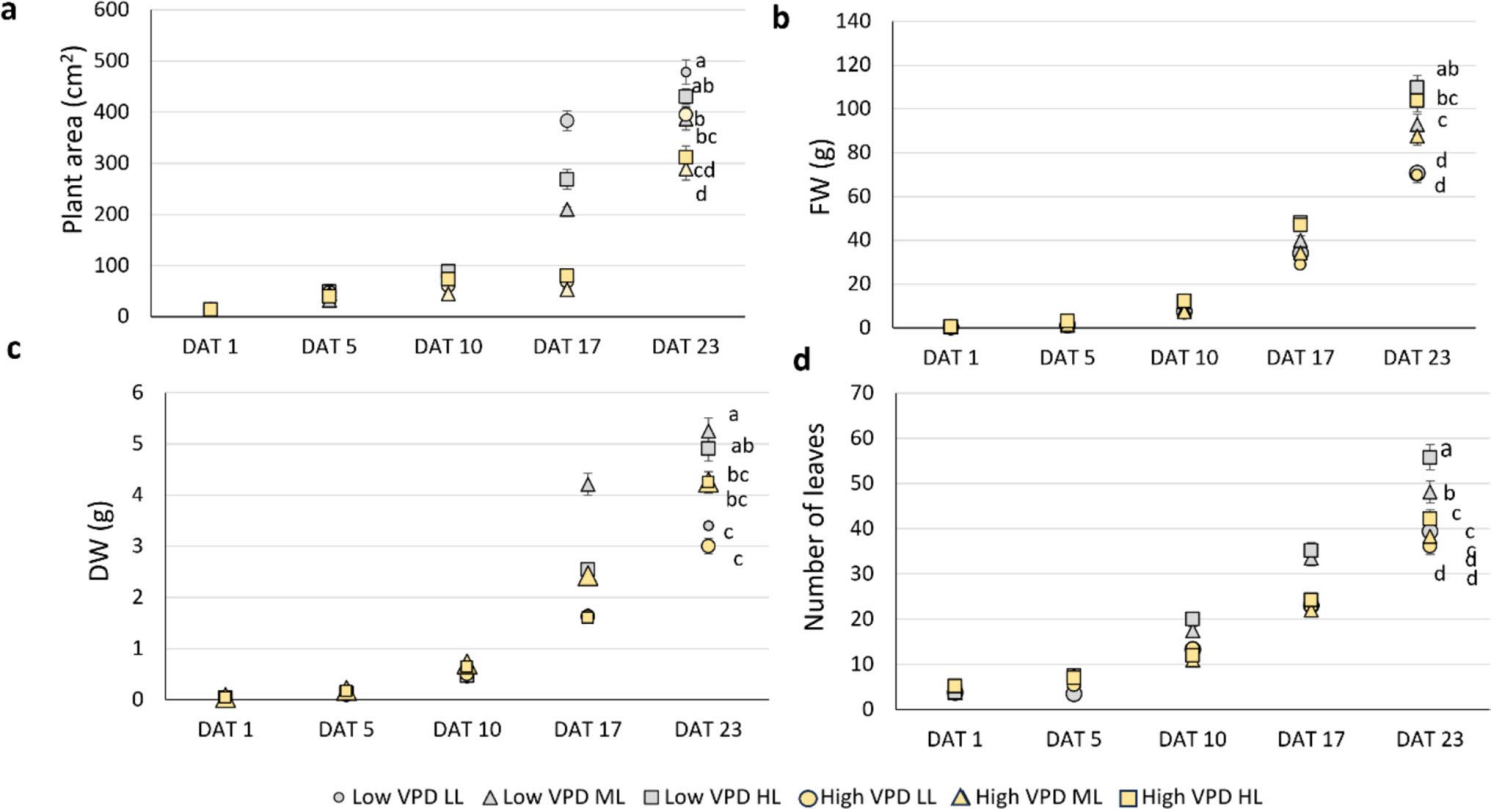
Growth curves in terms of plant area (**a**), fresh weight (FW, **b**), dry weight (DW, **c**) and number of leaves (**d**) in lettuces grown under low and high VPD at the three different DLIs (low light, LL; medium light, ML; high light, HL). Significance letters are shown only for the final harvest date; full statistical details for all time points are provided in the supplementary material

Growth dynamics in lettuce plants were significantly affected by both VPD and light intensity throughout the experimental period (Tables S1-S4; Fig. 2). Plant area (Fig. 2a) increased progressively over time, with the highest values recorded at DAT 23 under low VPD low light (LL), followed by high VPD LL and low VPD medium light (ML). Plants grown under high VPD in high light (HL) and ML conditions exhibited a 34–40% reduction in plant area compared to their low VPD counterparts. The fresh weight (FW; Fig. 2b) displayed a contrasting pattern, with plants under high light generally outperforming those grown under low light. Dry weight (DW; Fig. 2c) was similarly influenced by both environmental factors. The highest DW values were found under low VPD ML and HL, associated with greater production of leaves (Fig. 2d). Conversely, plants under both low and high VPD LL treatments consistently resulted in reduced DW and leaf production. Statistical significance is presented for the final harvest (23 DAT), while complete statistical results for earlier time points are available in Tables S1–S4.

### Leaf functional traits

Leaf functional traits were significantly influenced by both VPD and light intensity (Table 1). Leaf size generally decreased with increasing light intensity and was reduced under high VPD, except in high VPD LL plants. The largest leaves were observed in low VPD LL plants, while the smallest occurred at high VPD ML. Leaf mass per area (LMA) significantly increased with both light intensity and VPD, reaching the highest values in high VPD HL plants, more than doubling the value recorded under low VPD. The relative water content (RWC) was highest in low VPD ML plants, indicating optimal water status under moderate light and low evaporative demand. Conversely, high VPD HL plants exhibited the lowest RWC.

**Table 1:** Leaf traits in terms of leaf size, leaf mass per area (LMA), and relative water content (RWC) in lettuces grown under low and high VPD at the three DLIs (*LL* low light, *ML* medium light, *HL* high light)

	Leaf size (cm^2^)	LMA (mg cm^−2^)	RWC (%)
Low VPD LL	64.24 ± 0.98^a^	1.28 ± 0.04^e^	91.41 ± 1.30^ab^
Low VPD ML	55.73 ± 1.99^b^	1.66 ± 0.11^d^	93.84 ± 1.90^a^
Low VPD HL	50.89 ± 2.58^d^	2.36 ± 0.27^b^	81.53 ± 1.91^c^
High VPD LL	54.25 ± 3.64^b^	2.24 ± 0.09^c^	92.82 ± 1.96^a^
High VPD ML	43.10 ± 3.58^e^	2.17 ± 0.08^c^	91.45 ± 1.11^ab^
High VPD HL	51.99 ± 3.59^d^	3.13 ± 0.20^a^	68.20 ± 2.45^d^
*P*	*	*	*

Mean values and standard errors are shown (*n* = 6). Different letters indicate significant differences at *P* < 0.05 according to Tukey multiple range test. NS: non significant, *p* ≤ 0.001 (***), *p* ≤ 0.01 (**), *p* ≤ 0.05 (*)

### Photosynthetic performance

Photosynthetic performance varied significantly across treatments, reflecting interactive effects of VPD and light intensity on carbon assimilation and water use traits (Table 2). Net assimilation rate (A_N_) and stomatal conductance (g_S_) were enhanced under low VPD with values increasing along with light intensity. Transpiration rate (E) increased with light intensity, but higher E values were recorded at high VPD with the maximum transpiration occurring in high VPD HL plants. Along with the increase in photosynthetic rate under low VPD HL, these plants also showed the highest instantaneous water use efficiency (iWUE), together with low VPD LL. In contrast, iWUE declined sharply under high VPD, with the lowest values detected at high VPD HL.

**Table 2:** Leaf photosynthetic traits in terms of net assimilation rate (A_N_), stomatal conductance (g_s_), evapotranspiration (E), and instantaneous water use efficiency (iWUE) in lettuces grown under low and high VPD at the three DLIs (*LL* low light, *ML* medium light, *HL* high light)

	A_N_(µmol CO_2_m^−2^s^−1^)	g_s_(mol H_2_O m^−2^s^−1^)	E (mmol H_2_Om^−2^s^−1^)	iWUE (A_N_/E)
Low VPD LL	14.97 ± 0.46^d^	0.30 ± 0.004^c^	1.97 ± 0.20^f^	8.11 ± 0.01^a^
Low VPD ML	16.26 ± 0.02^c^	0.36 ± 0.002^b^	2.56 ± 0.01^e^	4.95 ± 0.02^c^
Low VPD HL	19.31 ± 0.03^a^	0.40 ± 0.004^a^	3.29 ± 0.02^d^	7.55 ± 0.01^ab^
High VPD LL	12.32 ± 0.01^f^	0.25 ± 0.015^d^	2.14 ± 0.00^ef^	5.77 ± 0.01^c^
High VPD ML	13.38 ± 0.01^e^	0.31 ± 0.010^c^	3.56 ± 0.01^c^	3.76 ± 0.01^d^
High VPD HL	15.15 ± 0.01^d^	0.31 ± 0.004^c^	4.41 ± 0.02^a^	3.44 ± 0.01^e^
*P*	**	***	*	*

Mean values and standard errors are shown (*n* = 10). Different letters indicate significant differences at *P* < 0.05 according to Tukey multiple range test. NS: non significant, *p* ≤ 0.001 (***), *p* ≤ 0.01 (**), *p* ≤ 0.05 (*)

### AN/I and AN/Ci response curves

The light response (A_N_/I) curves show the relationships between net-photosynthesis and light intensity levels for all the six treatments, with R^2^ ranging from 0.94 to 0.99 (Fig. S2), while the CO_2_ response (A/CO_2_) curves show the relationships between net photosynthesis and CO_2_ levels, with SSE from 0.02 to 0.12 (Fig. S3). Photosynthetic biochemical parameters derived from the A_N_/I and A_N_/CO_2_ response curves are summarized in Table 3. The maximum assimilation rate (A*max*) under low VPD HL significantly exceeded values of all other treatments, and decreased progressively under low VPD ML and LL. In contrast, plants under high VPD exhibited significantly lower A*max* values, particularly in high VPD LL and HL. The light compensation point (I*comp*) followed a similar trend, increasing by 10% from low VPD LL to low VPD HL. High VPD plants maintained relatively stable I*comp* values (~ 50–52).

**Table 3:** The variables calculated from the rectangular hyperbola Michaelis–Menten-based models (Lobo et al. 2013) to fit the net photosynthetic light–response curves: maximum assimilation rate (A*max*) and light compensation point (I*comp*) and the variables calculated from the non-linear curve fitting model (Sharkey et al. 2007) to fit the net photosynthetic CO_2_-response curves: maximum rate of Rubisco carboxylation (V*cmax*), maximum rate of electron transport (J*max*), in lettuces grown under low and high VPD at the three DLIs (*LL* low light, *ML* medium light, *HL* high light)

	A*max *(µmol CO_2_ m^−2^ s^−1^)	I*comp *(µmol photons m^−2^ s^−1^)	Vc*max *(µmol m^−2^ s^−1^)	J*max *(µmol m^−2^ s^−1^)
Low VPD LL	14.5 ± 0.01^e^	50.0 ± 0.12^c^	90 ± 0.31^c^	76 ± 0.11^f^
Low VPD ML	16.6 ± 0.04^c^	55.0 ± 0.18^b^	98 ± 0.40^b^	88 ± 0.21^e^
Low VPD HL	19.4 ± 0.06^a^	60.5 ± 0.21^a^	100 ± 0.03^a^	98 ± 0.03^c^
High VPD LL	14.1 ± 0.11^e^	50.0 ± 0.21^c^	84 ± 0.22^d^	111 ± 0.09^b^
High VPD ML	16.2 ± 0.09^c^	52.2 ± 0.09^bc^	84 ± 0.11^d^	115 ± 0.36^b^
High VPD HL	15.4 ± 0.21^d^	50.9 ± 0.12^c^	113 ± 0.29^a^	113 ± 0.21^b^
*P*	***	**	***	***

Mean values and standard errors are shown (*n* = 10). Different letters indicate significant differences at *P* < 0.05 according to Tukey multiple range test. NS: non significant, *p* ≤ 0.001 (***), *p* ≤ 0.01 (**), *p* ≤ 0.05 (*)

The carboxylation capacity of Rubisco (V*cmax*) mirrored A*max* trends under low VPD, increasing with light availability. Under high VPD; however V*cmax* remained lower in LL and ML, but increased sharply under HL, matching the value in low VPD HL. Notably, the maximum electron transport rate (J*max*) was consistently higher in high VPD plants compared to their low VPD counterparts, regardless of light intensity and with no significant differences among them.

### Plant anatomy

#### Mesophyll anatomical traits

Mesophyll anatomy was significantly influenced by both VPD and light intensity, with multiple traits exhibiting distinct acclimation patterns across treatments (Table 4). Upper epidermis thickness was significantly increased at high VPD HL (29%), distinguishing it from all other conditions. In contrast, no differences were found in lower epidermis thickness. Palisade thickness increased with light, independent of VPD, thus peaking in both low VPD HL and high VPD HL. Spongy thickness showed an inverse trend, reaching higher values in both low VPD LL and high VPD LL. Total mesophyll thickness peaked in high VPD ML, with high VPD HL and low VPD LL. In high VPD HL and low VPD LL the enhanced mesophyll was accompanied by increased intercellular space percentages. Mesophyll gas phase conductance (g_ias_) decreased sharply under high VPD, particularly in HL plants. The highest values were observed in low VPD LL.

**Table 4:** Leaf mesophyll anatomical traits in terms of upper epidermis thickness, palisade thickness, spongy thickness, lower epidermis thickness, mesophyll thickness, percentage of intercellular spaces, mesophyll intercellular air space conductance of the gas phase (g_ias_) in lettuces grown under low and high VPD at the three DLIs (*LL* low light, *ML* medium light, *HL* high light)

	Upper epidermis thickness (µm)	Palisade thickness (µm)	Spongy thickness (µm)	Lower epidermis thickness (µm)	Mesophyll thickness (µm)	Intercellular spaces (%)	g_ias_(S m^₋2^)
Low VPD LL	8.70 ± 0.26^b^	39.66 ± 0.65^d^	104.07 ± 5.15^a^	8.31 ± 1.34^a^	160.7 ± 5.15^ab^	13.96 ± 0.54^a^	0.42 ± 0.03^a^
Low VPD ML	8.99 ± 0.25^b^	51.33 ± 1.54^b^	85.60 ± 2.52^c^	7.05 ± 1.28^a^	152.9 ± 3.16^b^	9.42 ± 0.21^c^	0.38 ± 0.01^b^
Low VPD HL	8.14 ± 0.27^b^	56.13 ± 1.23^a^	77.94 ± 1.58^c^	6.65 ± 2.21^a^	148.8 ± 2.20^b^	9.30 ± 0.15^c^	0.27 ± 0.01^d^
High VPD LL	8.56 ± 0.26^b^	35.75 ± 0.59^d^	105.11 ± 3.72^a^	7.57 ± 2.29^a^	156.9 ± 3.70^b^	10.97 ± 0.36^b^	0.31 ± 0.03^c^
High VPD ML	8.48 ± 0.25^b^	46.75 ± 1.05^c^	109.20 ± 3.15^a^	7.97 ± 1.28^a^	172.4 ± 3.72^a^	9.66 ± 1.29^c^	0.35 ± 0.04^c^
High VPD HL	11.33 ± 0.42^a^	52.46 ± 1.34^a^	88.21 ± 3.87^b^	9.43 ± 2.35^a^	161.4 ± 3.58^ab^	13.55 ± 1.10^a^	0.16 ± 0.04^d^
*P*	**	**	***	NS	*	**	**

Mean values and standard errors are shown (*n* = 10). Different letters indicate significant differences at *P* < 0.05 according to Tukey multiple range test. NS: non significant, *p* ≤ 0.001 (***), *p* ≤ 0.01 (**), *p* ≤ 0.05 (*)

#### Stomatal and vein anatomical traits

**Fig. 3:**
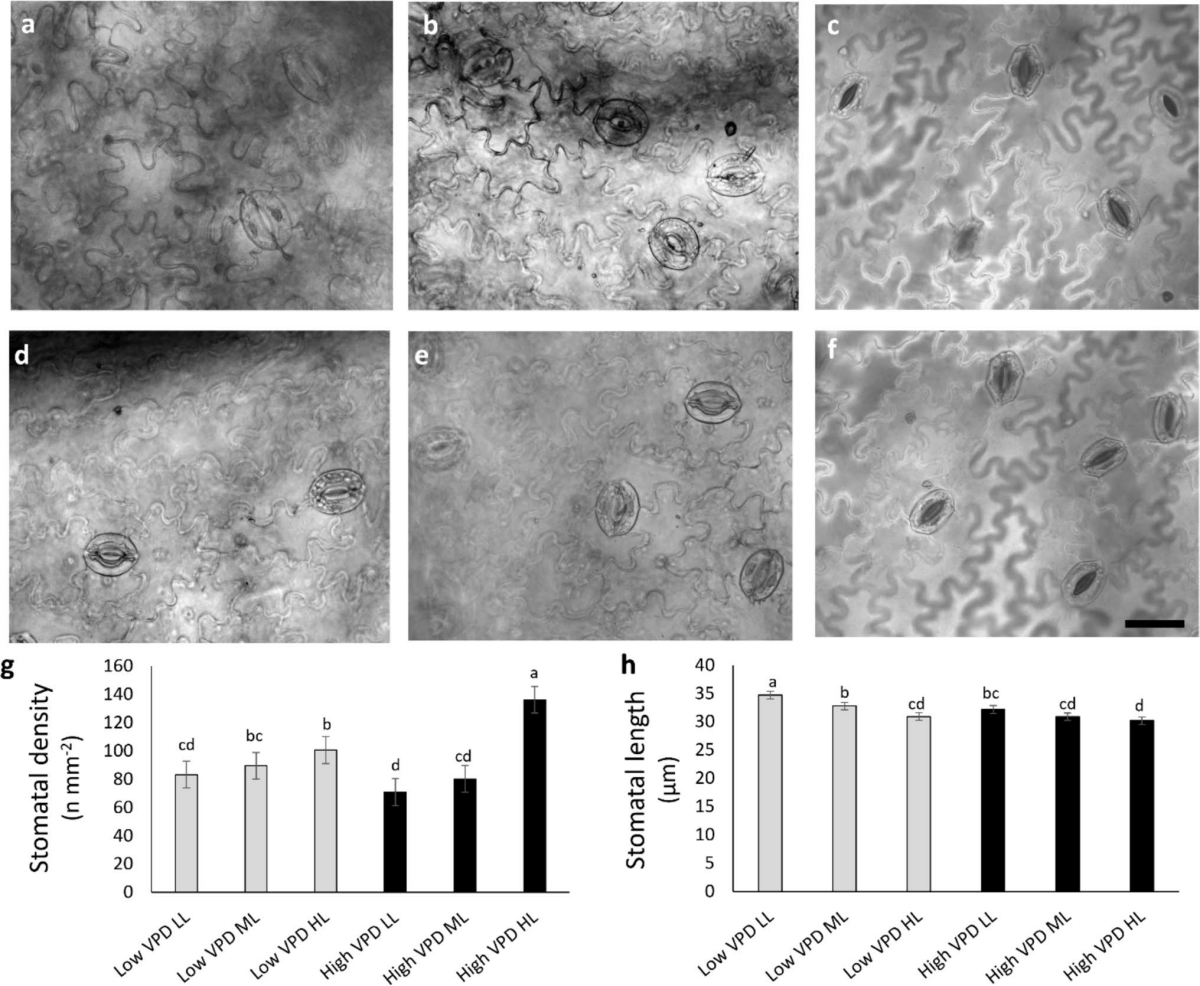
Light microscopy views of leaf epidermal peels showing stomata (**a**-**f**) for lettuces grown under low VPD (**a-c**) and high VPD (**d**-**f**) at the three DLIs (low light, LL; medium light, ML; high light, HL). Images are at the same magnification and scale bar is 40 μm. **g**, **h** Stomata density (**g**) and stomata length (**h**). Mean values and standard errors (*n* = 10) are shown. Different letters correspond to significantly different values according to Tukey post hoc test

Microscopy observations and quantitative analyses revealed significant effects of VPD and light on stomatal traits (Fig. 3). high VPD HL developed more stomata per mm^2^, significantly exceeding all other treatments (overall 40% increments) (Fig. 3g). These plants also presented the smallest stomata (Fig. 3h). In contrast, the lowest densities were observed under high VPD LL, high VPD ML, and low VPD LL. Guard cell length tended to decrease with increasing light in both VPD conditions.

**Fig. 4:**
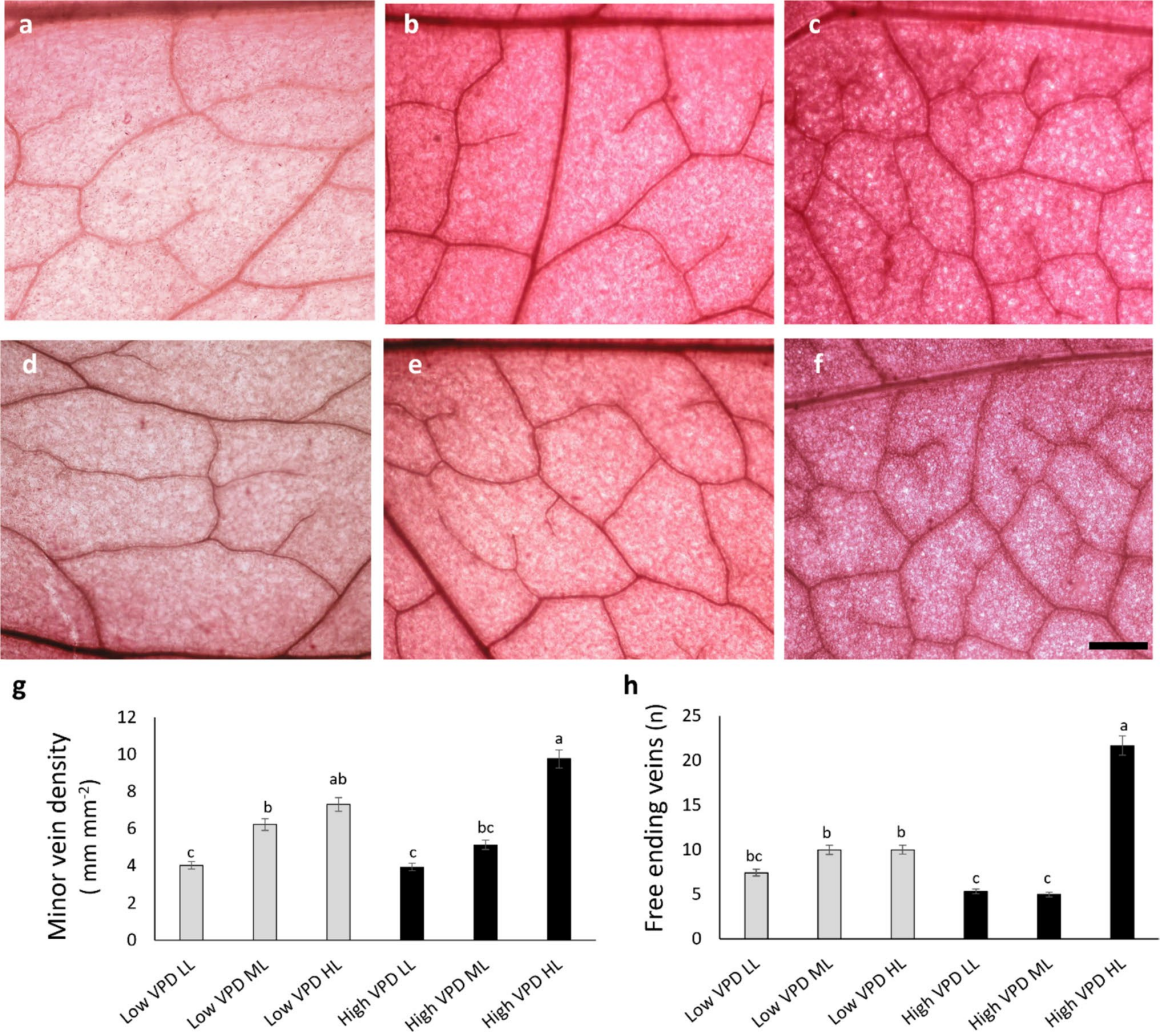
Light microscopy views of leaf epidermal peels showing veins (**a**-**f**) for lettuces grown under low VPD (**a**-**c**) and high VPD (**d**-**f**) at the three DLIs (low light, LL; medium light, ML; high light, HL). Images are at the same magnification and scale bar is 20 μm. **g**, **h** Minor vein density (VLA; **g**) and free endings veins (FEV; **h**). Mean values and standard errors (*n* = 10) are shown. Different letters correspond to significantly different values according to Tukey post hoc test

Vein patterning was also affected by VPD and light intensity (Fig. 4). Consistently with stomata traits, minor vein density (Fig. 4g) was markedly enhanced under high VPD HL, with values more than doubling those of low VPD LL plants. A similar pattern was observed for the number of free-ending veins (Fig. 4h), which was significantly higher in high VPD HL leaves compared to all the other treatments (50% enhancement compared to low VPD HL).

#### Chloroplast distribution and carbon diffusion parameters

**Fig. 5:**
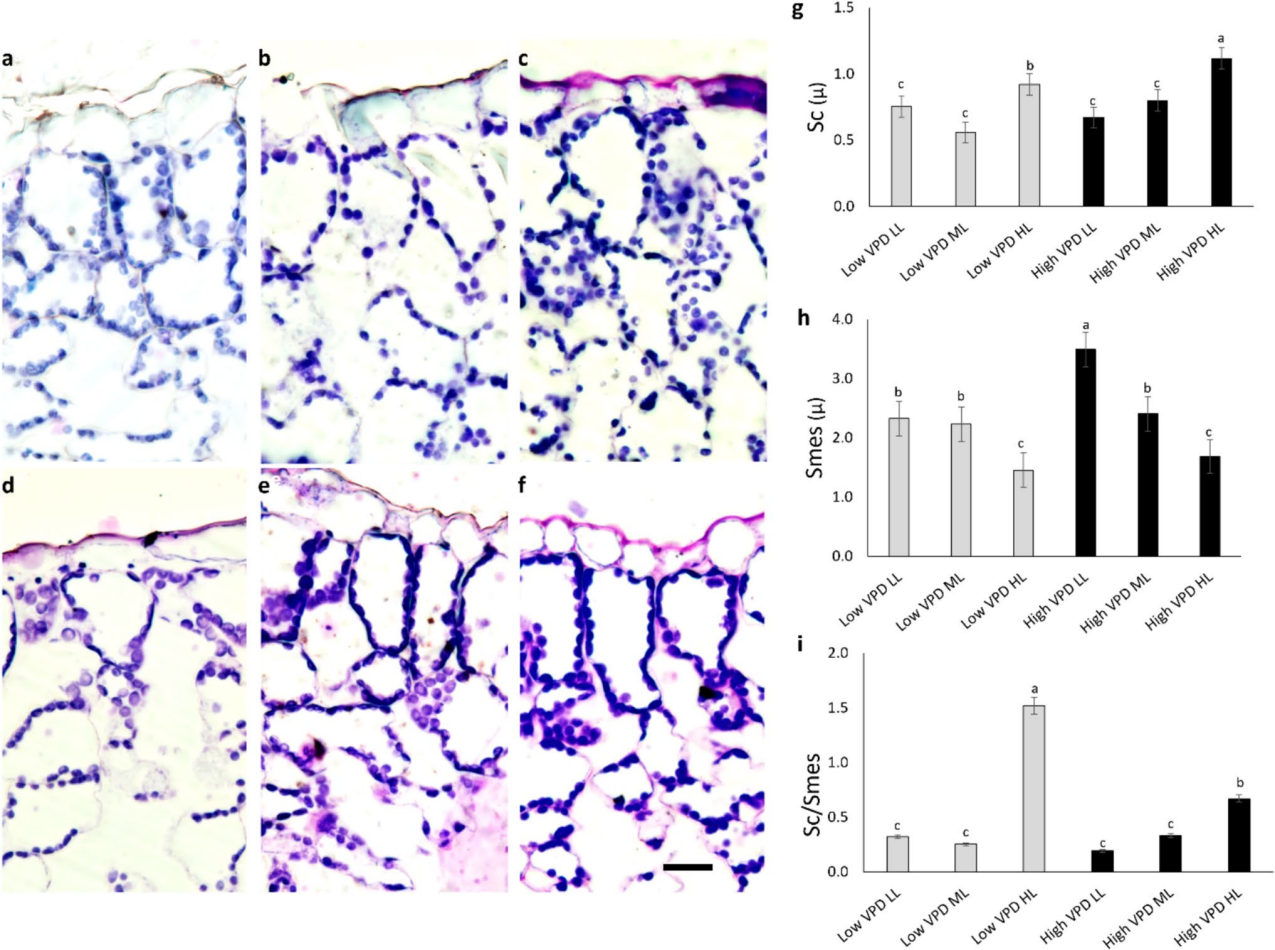
Light microscopy views of leaf lamina cross sections focusing on chloroplasts in palisade parenchyma (**a-f**) for lettuces grown under low VPD (**a**-**c**) and high VPD (**d**-**f**) at the three DLIs: low light, LL (**a**, **d**); medium light, ML (**b**, **e**); high light, HL (**c**, **f**). Images are at the same magnification and scale bar is 20 μm. **g**-**i** The area of the chloroplast surfaces facing the intercellular space per unit leaf area (Sc; **g**), the intercellular space per unit leaf area (Smes; **h**), and their ratio (Sc/Smes; **i**). Mean values and standard errors are shown (*n* = 10). Different letters correspond to significantly different values according to Tukey post hoc test

Cross sections, stained to visualize chloroplast arrangement in the mesophyll, revealed significant anatomical differences (Fig. 5a-f). The surface area of chloroplast exposure to intercellular airspace (Sc) (Fig. 5g) increased with light and VPD, peaking at high VPD HL, which also exhibited compact palisade arrangements (Fig. 5f). In contrast, the mesophyll surface area (Smes) (Fig. 5h) was highest at high VPD LL. As a consequence, the ratio Sc:Smes (Fig. 5i), a proxy for the anatomical limitation to CO₂ diffusion, was lowest at low and moderate light intensities, and reached its maximum value under low VPD HL, followed by high VPD HL.

#### Multivariate analysis

**Fig. 6:**
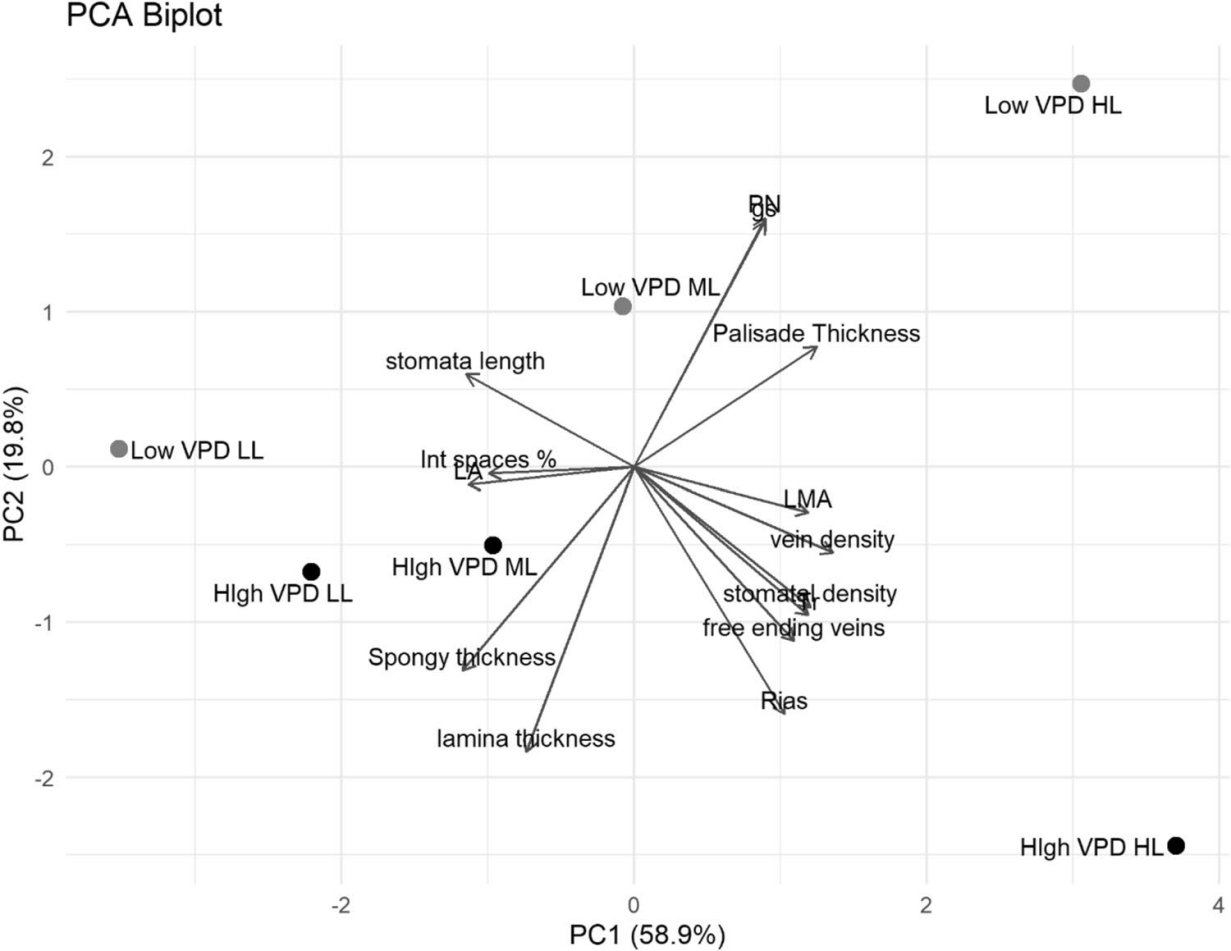
Principal component analysis (PCA) of anatomical and physiological traits in lettuce plants grown under low and high VPD at three different DLIs (*LL* low light, *ML* medium light, *HL* high light). The plot displays the distribution of treatments in the space defined by the first two principal components PC1 (58.9%) and PC2 (19.8%)

A principal component analysis (PCA) was performed to explore the multivariate relationships among anatomical and physiological traits across light and VPD treatments (Fig. 6). The first two principal components explained a combined 78.7% of the total variance (PC1: 58.9%; PC2: 19.8%). PC1 was mainly explained by the variability among light intensity treatments, while PC2 was mainly associated with VPD. High VPD HL plants, standing alone in the lower-right quadrant, were strongly positively associated with traits, such as LMA, vein, and stomatal density, and the number of free-ending veins—indicative of a stress-induced anatomical adjustment. In contrast, high VPD LL and ML treatments were clustered on the opposite end of PC1, closely aligned with lamina and spongy mesophyll thickness, and negatively associated with traits indicative of carbon assimilation efficiency. The low VPD HL treatment, positioned in the upper-right quadrant, was strongly associated with as net assimilation rate (A_N_), palisade tissue thickness and stomatal conductance (g_s_).

**Fig. 7:**
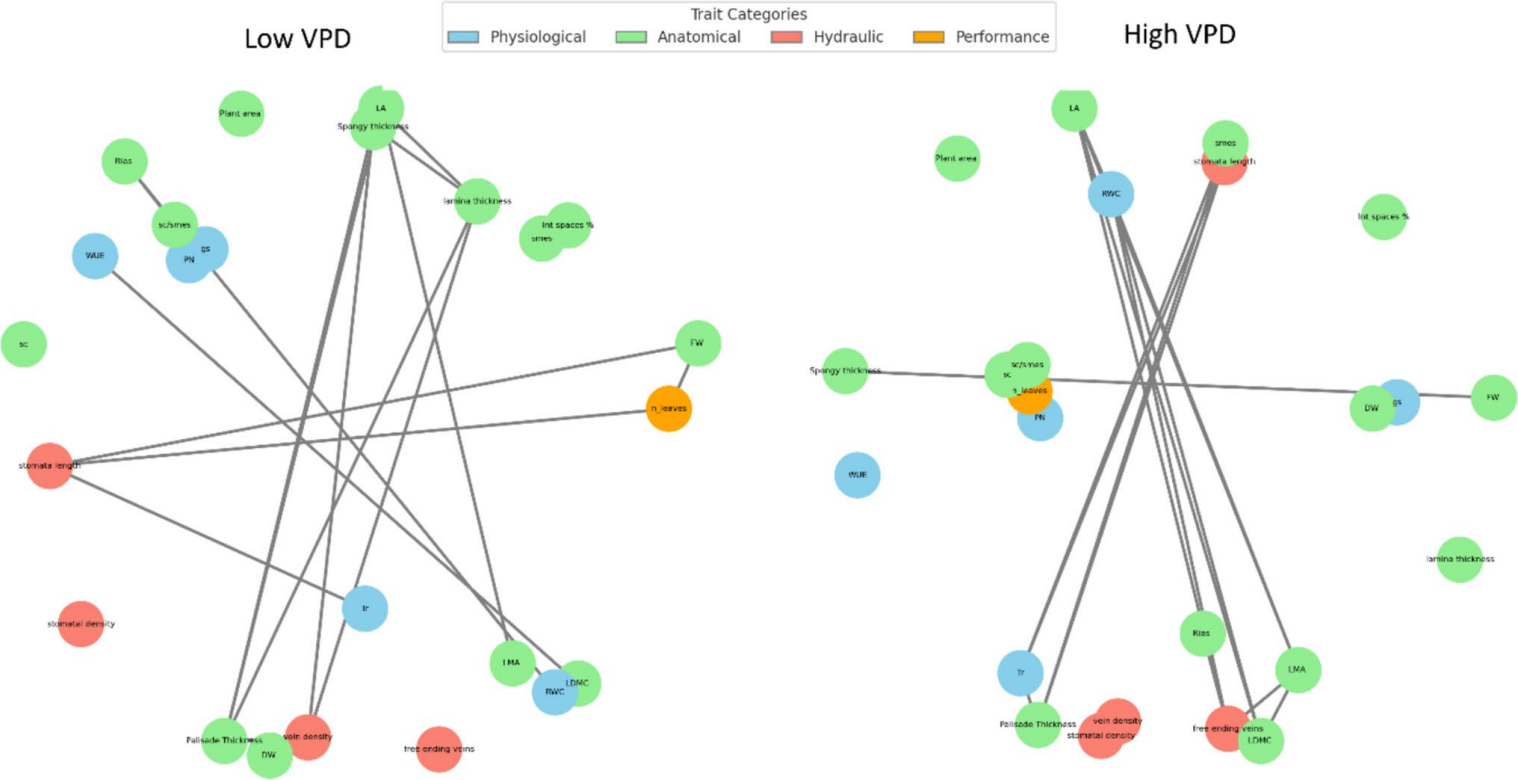
Trait correlation networks of lettuce plants grown under low and high VPD conditions. Networks display significant Spearman correlations (*P* ≤ 0.05) among physiological (blue), anatomical (green), hydraulic (red), and performance (orange) traits. Thicker edges represent stronger correlations

To explore the coordination among anatomical, physiological, hydraulic, and performance-related traits under contrasting environmental conditions, trait network analysis was conducted under low and high VPD (Fig. 7). The network structure differed markedly between the two VPD regimes. Under low VPD, the network was composed of 24 nodes and 21 edges, with an average degree of 1.75 and a network density of 0.076 (Table S5). The average clustering coefficient was 0.36, indicating moderate local connectivity among traits. In contrast, the high VPD network displayed a denser and more interconnected structure, with 24 nodes and 24 edges, an average degree of 2.00, and higher network density (0.087) and clustering coefficient (0.46).

The high VPD condition exhibited a greater number of connections among anatomical and hydraulic traits (e.g., vein density, stomatal density, LMA), as well as tighter associations between structural and functional traits (e.g., vein traits and PN or gs). Notably, free-ending veins and vein density emerged as central nodes in the high VPD network, linked to both anatomical and performance-related variables. In contrast, under low VPD, coordination was less centralized, with clusters of associations limited to specific subsets of traits (e.g., mesophyll traits with gas exchange, and biomass with stomatal traits).

## Discussion

This study provides compelling evidence that ‘Salanova’ lettuce exhibits strong phenotypic plasticity in response to simultaneous variation in vapor pressure deficit (VPD) and light intensity. While we originally hypothesized that the combination of high VPD and high DLI would negatively impact mesophyll anatomical development and suppress physiological performance, our findings challenge this assumption. Specifically, plants grown under high VPD and high light conditions showed remarkable anatomical plasticity, suggesting a robust capacity to acclimate to moderately challenging environments through anatomical adjustment.

Although light-induced anatomical responses have been well documented in leafy crops, our results advance the understanding of how light intensity interacts with atmospheric VPD to modulate internal leaf development. Some traits followed expected patterns of light acclimation: for instance, plants growing at low VPD under low light (LL) developed the largest leaf area (64.24 cm^2^), but their overall biomass accumulation and photosynthetic performance were inferior to those of plants in conditions of low VPD and medium (ML) or high (HL) light. This aligns with established trade-offs between light capture and efficiency: plants under low irradiance often allocate resources to maximize leaf expansion at the expense of tissue density and carbon gain, indicative of a shade-acclimation strategy (Valladares and Niinemets 2008; Niinemets 2010). The large lamina size under low light likely results from increased cell and tissue expansion, which can influence areal traits such as stomatal and vein density through geometric dilution. This passive effect, as described by Carins Murphy et al. (2012), may explain part of the reduced densities observed under LL, independently of active regulation. In contrast, HL plants displayed thicker leaves, higher LMA, and increased palisade parenchyma, as typical adaptations to high irradiance to maximize carbon gain and protect against photodamage (Wang et al. 2016). Similar light-induced mesophyll adaptations have been reported in lettuce and spinach under variable DLI regimes (Wang et al. 2016), corroborating that light environments shape key anatomical features such as palisade thickness and LMA. Our data notably revealed that these traits were even more pronounced under high VPD, suggesting that evaporative demand amplifies anatomical investment. Indeed, plants exposed to high VPD allocate more resources to the development of internal structures that support water and gas transport. For example, high VPD plants exhibited increased spongy mesophyll thickness and intercellular airspace percentage, potentially enhancing CO₂ diffusion efficiency. In parallel, higher stomatal and vein densities under high VPD and high light likely help maintain physiological function under elevated transpiration rates. Increased LMA in low VPD HL plants was primarily linked to palisade thickening, a common response to high light. In contrast, under high VPD, the increase in LMA was likely associated with a more developed spongy mesophyll, despite the concurrent increase in intercellular airspace. This configuration, compared to their low VPD counterparts, may represent an anatomical strategy to enhance internal CO₂ diffusion under high evaporative demand (Carins Murphy et al. 2012; Tholen et al. 2012). Furthermore, chloroplast repositioning emerged as a key acclimation strategy. Under high DLI, plants exhibited increased Sc (chloroplasts facing the intercellular space) under both VPD conditions, reflecting a typical acclimation strategy to enhance CO₂ uptake under elevated photosynthetic demand. This aligns with findings by Oguchi et al. (2003) who demonstrated that, in *Chenopodium album*, the exposure to high light triggers chloroplast repositioning toward the cell walls adjacent to intercellular airspaces, effectively increasing the surface area available for CO₂ uptake and enhancing diffusion efficiency, without requiring expansion of the mesophyll structure. However, unlike the findings in *C. album* reported by Oguchi et al. (2003), where high light exposure increased the mesophyll surface area (Smes), our data show a reduction in Smes under high light conditions. This suggests that in our lettuces, physical expansion of the mesophyll is spatially constrained, possibly due to intrinsic anatomical limitations such as compact mesophyll organization or limited intercellular space development. Consequently, rather than increasing mesophyll surface area, lettuce appears to optimize internal CO₂ diffusion by repositioning chloroplasts closer to intercellular airspaces. This pattern of compensatory chloroplast redistribution, rather than mesophyll expansion, has also been reported in dense-leaved species by Xiong et al. (2023).

Importantly, across treatments, plants under low VPD consistently outperformed those under high VPD in biomass accumulation, gas exchange, and water use efficiency, also developing a more “efficient” anatomical organization of tissues, supporting the broader literature indicating that reduced evaporative demand fosters better physiological conditions for crop productivity (Grossiord et al. 2020; Xiong and Flexas 2020). For instance, a consistently higher stomatal and vein densities under low VPD, typically associated with better coordination of hydraulic and photosynthetic traits, may also be the consequence of the reduced passive leaf expansion compared to high VPD plants, given that the relation between leaf lamina expansion and stomata density has been observed in many species over recent years (Carins Murphy et al. 2012, 2016; Du et al. 2020; Amitrano et al. 2021a,b). A key observation of this study is that under high VPD HL conditions this trend is not maintained. Although these plants operated under the most demanding microclimatic conditions in our experimental design (highest light, highest evaporative demand), they developed anatomical configurations that in several aspects resembled or even exceeded those observed under low VPD HL. high VPD HL plants exhibited increased stomatal density and reduced stomatal size, a combination known to optimize gas exchange regulation (Haworth et al. 2023; Lawson and Leaky 2024). Similarly, a coordination between stomatal and vein development was evident, with high VPD HL plants doubling minor vein density and increasing by 50% free-ending veins suggesting enhanced investment in water transport capacity to meet elevated transpirational demand (Brodribb and Jordan 2011). Recent evidence has shown that high vein density and increased presence of free-ending veins contribute to improved hydraulic redundancy providing photosynthetic stability, particularly under environmentally demanding conditions (Scoffoni et al. 2011; Sack and Scoffoni 2013). However, reductions in mesophyll gas phase conductance (g_ias_) under high VPD indicate that the expanded intercellular airspace—while potentially beneficial for CO₂ diffusion—may also introduce resistance to gas flow when structural thresholds are exceeded. This indicates that beyond a certain point, increased porosity may introduce resistance to gas flow, possibly due to increased path length or less efficient diffusion geometry (Amitrano et al. 2023). These patterns highlight the complexity of internal diffusion dynamics and underscore that structural responses to VPD and light are not always functionally synergistic, and may become constrained or even maladaptive under combined environmental challenges.

Because of these anatomical plasticity, high VPD HL plants maintained intermediate A_N_ across treatments and upregulated V*cmax*, also suggesting a degree of biochemical compensation. J*max* values were also consistently higher in high VPD plants under HL, indicating enhanced electron transport capacity. Despite reductions in iWUE (instantaneous water use efficiency) in high VPD HL plants, these values did not significantly differ from high VPD ML plants. These trends suggest the activation of compensatory structural and biochemical strategies that buffer productivity losses and partially sustain photosynthetic function under severe evaporative demand, reinforcing the idea that acclimation in these plants was primarily driven by morpho-anatomical plasticity.

Principal component analysis and trait network further corroborated these dynamics. Indeed, the PCA clearly separated high VPD HL from other treatments, associating it with traits promoting anatomical acclimation, while low VPD HL clustered with traits supporting carbon assimilation. Moreover, the shift in network structure under high VPD is biologically meaningful: the higher average degree (2.00 vs. 1.75) indicates a greater number of trait interconnections per node, suggesting stronger coordination among anatomical, physiological, and hydraulic traits. The increase in network density (0.087 vs. 0.076) implies a more integrated trait system, where more relationships among traits are expressed relative to the maximum possible. Finally, the higher clustering coefficient (0.46 vs. 0.36) reflects a greater tendency for interconnected traits to form local sub-networks, likely representing tightly regulated trait modules under more demanding environmental conditions. These structural differences support the view that high VPD triggers a more canalized and functionally interdependent configuration of leaf traits, possibly enhancing physiological stability to maintain function under challenging environmental conditions (Li et al. 2021; Haworth et al. 2023).

## Conclusion

This study demonstrates how ‘Salanova’ lettuce orchestrates a suite of structural and functional adjustments in response to simultaneous fluctuations in VPD and light. We hypothesized that different irradiances could alter plant responses to VPD, modulating anatomical and photosynthetic traits. Moreover, we hypothesized that the combination of the highest irradiance and VPD level (high VPD HL) would create an excessively dry microenvironment surrounding the plants, negatively affecting mesophyll development and, therefore, plant physiological behavior. Unexpectedly, high VPD HL lettuces showed a high degree of anatomical plasticity in stomatal, mesophyll, and vascular traits revealing a finely tuned system for balancing carbon acquisition and water conservation. While anatomical investments under high VPD HL may not always translate into functional efficiency, they reflect an attempt to preserve physiological integrity. This anatomical plasticity is crucial in the context of climate change and should be further explored in other species. Our results could also inform on sustainable indoor cultivation strategies and enhance our understanding of lettuce’s ecological adaptations to increasingly prevalent environmental challenges associated with climate change. Further studies on other crop species and long-term exposure experiments are warranted to delineate the limits of anatomical acclimation and identify thresholds beyond which plasticity becomes maladaptive.

## Supplementary Information

Below is the link to the electronic supplementary material.

Supplementary file1 (DOCX 722 KB)

## Data Availability

The data supporting the findings of this study are available from the corresponding author, upon reasonable request.

## Abbreviations

DAT: Day after transplanting
DLI: Daily light integral
LMA: Leaf mass per area
VPD: Vapor pressure deficit
