# Correction: Leaf anatomical traits shape lettuce physiological response to vapour pressure deficit and light intensity

**DOI:** 10.1007/s00425-025-04831-w

**Published:** 2025-09-29

**Authors:** Chiara Amitrano, Murat Kacira, Carmen Arena, Stefania De Pascale, Veronica De Micco

**Affiliations:** 1 Department of Agricultural Sciences, University of Naples Federico II, Portici, Naples, Italy; 2 Department of Biosystems Engineering, University of Arizona, Tucson, AZ USA; 3 Department of Biology, University of Naples Federico II, Naples, Italy

**Correction: Planta (2025) 262:48** 10.1007/s00425-025-04774-2

The article Leaf anatomical traits shape lettuce physiological response to vapour pressure deficit and light intensity, written by Chiara Amitrano, Murat Kacira, Carmen Arena, Stefania De Pascale, Veronica De Micco, was originally published under exclusive license to ‘The Author(s), under exclusive licence to Springer-Verlag GmbH Germany, part of Springer Nature 2025’. As a result of the subsequent decision to publish the article under the open access model, the article’s copyright notice was changed on 15 September, 2025 to © The Author(s) 2025 and the article is now distributed under a Creative Commons Attribution 4.0 International License, which permits use, sharing, adaptation, distribution and reproduction in any medium or format, as long as you give appropriate credit to the original author(s) and the source, provide a link to the Creative Commons licence, and indicate if changes were made. The images or other third party material in this article are included in the article's Creative Commons licence, unless indicated otherwise in a credit line to the material. If material is not included in the article's Creative Commons licence and your intended use is not permitted by statutory regulation or exceeds the permitted use, you will need to obtain permission directly from the copyright holder. To view a copy of this licence, visit http://creativecommons.org/licenses/by/4.0/.

Open access funding provided by Università degli Studi di Napoli Federico II within the CRUI-CARE Agreement.

The original article has been corrected.

