# Supplementary material for: Leaf anatomical traits shape lettuce physiological response to vapor pressure deficit and light intensity
Source: Planta. 2025 Jul 13;262(2):48. doi: 10.1007/s00425-025-04774-2 (PMC12479579; doi:10.1007/s00425-025-04774-2)

**Title:** Leaf anatomical traits shape lettuce physiological response to vapor pressure deficit and light intensity.

Chiara Amitrano^1*^, Murat Kacira^2^, Carmen Arena^3^, Stefania De Pascale^1^, Veronica De Micco^1^

Table S1. Plant area of lettuces grown under low and high VPD at the three DLIs (low light, LL; medium light, ML; high light, HL). Mean values and standard errors are shown (*n* = 5). Different letters indicate significant differences at *P*<0.05 according to Tukey multiple range test.

| **Plant Area**  (cm^2^) | **5**  DAT | **10**  DAT | | **17**  DAT | | **23**  DAT |
| --- | --- | --- | --- | --- | --- | --- |
| Low VPD LL | 40.02 ± 0.04^c^ | | 75.62 ± 0.27^c^ | | 383.68 ± 3.65^a^ | 478.44 ± 2.04^a^ |
| Low VPD ML | 32.00 ± 1.73^e^ | | 83.97 ± 5.09^b^ | 209.74 ± 4.13^c^ | | 386.72 ± 2.28^bc^ |
| Low VPD HL | 48.80 ± 0.93^a^ | | 87.66 ± 9.09^a^ | 268.51 ± 1.88^b^ | | 430.22 ± 1.39^ab^ |
| High VPD LL | 47.00 ± 0.86^ab^ | | 60.57 ± 0.19^d^ | 68.75 ± 3.96^e^ | | 395.05 ± 1.41^b^ |
| High VPD ML | 45.20 ± 2.65^b^ | | 44.26 ± 0.11^e^ | 53.27 ± 1.45^d^ | | 289.14 ± 2.19^e^ |
| High VPD HL | 39.83 ± 6.38^d^ | | 73.00 ± 2.14^c^ | 79.49 ± 3.35^d^ | | 311.92 ± 2.27^cd^ |
| *P* | * | | * | * | | ** |

Table S2. Fresh weight of lettuces grown under low and high VPD at the three DLIs (low light, LL; medium light, ML; high light, HL). Mean values and standard errors are shown (*n* = 5). Different letters indicate significant differences at *P*<0.05 according to Tukey multiple range test.

| **FW**  (g) | **5**  DAT | **10**  DAT | **17**  DAT | **23**  DAT |
| --- | --- | --- | --- | --- |
| Low VPD LL | 1.22 ± 0.10^d^ | 7.56 ± 0.44^c^ | 34.13 ± 1.75^c^ | 70.75 ± 3.48^d^ |
| Low VPD ML | 1.26 ± 0.10^fd^ | 8.61 ± 0.97^b^ | 40.00 ± 2.40^b^ | 92.92 ± 5.19^c^ |
| Low VPD HL | 1.99 ± 0.01^c^ | 11.71 ± 0.95^a^ | 48.13 ± 1.14^a^ | 109.83 ± 1.84^ab^ |
| High VPD LL | 2.35 ± 0.20^b^ | 12.52 ± 0.22^a^ | 28.84 ± 0.33^d^ | 69.67 ± 2.87^d^ |
| High VPD ML | 1.53 ± 0.01^d^ | 7.84 ± 1.00^c^ | 34.32 ± 1.15^c^ | 87.67 ± 1.15^c^ |
| High VPD HL | 3.11 ± 0.10^a^ | 12.32 ± 0.28^a^ | 46.90 ± 4.35^ab^ | 103.75 ± 0.86^bc^ |
| *P* | ** | ** | ** | * |

Table S3. Dry weight (DW) of lettuces grown under low and high VPD at the three DLIs (low light, LL; medium light, ML; high light, HL). Mean values and standard errors are shown (*n* = 5). Different letters indicate significant differences at *P*<0.05 according to Tukey multiple range test.

| **DW**  (g) | **5**  DAT | **10**  DAT | **17**  DAT | **23**  DAT |
| --- | --- | --- | --- | --- |
| Low VPD LL | 0.11 ± 0.01^a^ | 0.43 ± 0.03^c^ | 1.67 ± 0.09^c^ | 3.40 ±0.15^c^ |
| Low VPD ML | 0.16 ± 0.02^a^ | 0.62 ± 0.04^b^ | 4.21 ± 0.02^a^ | 5.25 ± 0.49^a^ |
| Low VPD HL | 0.12 ± 0.01^a^ | 0.47 ± 0.04^c^ | 2.54 ± 0.14^b^ | 4.91 ± 0.24^ab^ |
| High VPD LL | 0.09 ± 0.01^a^ | 0.49 ± 0.02^c^ | 1.62 ± 0.23^c^ | 3.00 ± 0.13^c^ |
| High VPD ML | 0.18 ± 0.01^a^ | 0.69 ± 0.05^ab^ | 2.42 ± 0.41^b^ | 4.25 ± 0.11^bc^ |
| High VPD HL | 0.17 ± 0.01^a^ | 0.64 ± 0.02^b^ | 1.60 ± 0.05^c^ | 4.25 ± 0.28^bc^ |
| *P* | NS | * | * | * |

Table S4. Number of leaves of lettuces grown under low and high VPD at the three DLIs (low light, LL; medium light, ML; high light, HL). Mean values and standard errors are shown (*n* = 5). Different letters indicate significant differences at *P*<0.05 according to Tukey multiple range test.

| **Number of leaves** | **5**  DAT | **10**  DAT | **17**  DAT | **23**  DAT |
| --- | --- | --- | --- | --- |
| Low VPD LL | 3.50 ± 0.50^d^ | 13.33 ± 0.17^b^ | 23.00 ± 0.01^bc^ | 39.50 ± 1.15^cd^ |
| Low VPD ML | 6.50 ± 0.50^b^ | 17.50 ± 0.50^ab^ | 33.50 ± 1.55^a^ | 48.17 ± 1.25^b^ |
| Low VPD HL | 7.50 ± 0.51^a^ | 20.00 ± 0.01^a^ | 35.25 ± 1.84^a^ | 55.83 ± 0.54^a^ |
| High VPD LL | 5.50 ± 0.50^c^ | 13.50 ± 0.50^b^ | 22.75 ± 1.11^c^ | 36.17 ± 2.14^d^ |
| High VPD ML | 7.50 ± 0.50^a^ | 11.00 ± 1.00^c^ | 22.00 ± 1.08^c^ | 38.33 ± 0.92^cd^ |
| High VPD HL | 7.00 ± 0.01^bb^ | 12.00 ± 1.00^c^ | 24.25 ± 0.63^b^ | 42.17 ± 1.70^c^ |
| *P* | ** | * | ** | ** |

Table S5. Summary of trait network metrics under Low and High VPD conditions.
Each network includes 24 trait nodes, with edges representing significant Spearman correlations ( *P* ≤ 0.05). The average degree, density, and clustering coefficient provide insight into the connectivity and integration of traits within each environmental condition.

|  | **Number of nodes** | **Number of edges** | **Average**  **degree** | **Density** | **Average clustering** |
| --- | --- | --- | --- | --- | --- |
| Low VPD | 24 | 21 | 175 | 7.6 E+15 | 3.6 E+16 |
| High VPD | 24 | 24 | 20 | 8.7 E+15 | 4.5 E+16 |

**Figure legends**

**Fig. S1** Environmental parameters in terms of Temperature (T) and relative humidity (RH) (**a**), CO_2_ concentration (**b**), electrical conductivity and pH (**c**), dissolved oxygen (**d**) for low and high VPD conditions.


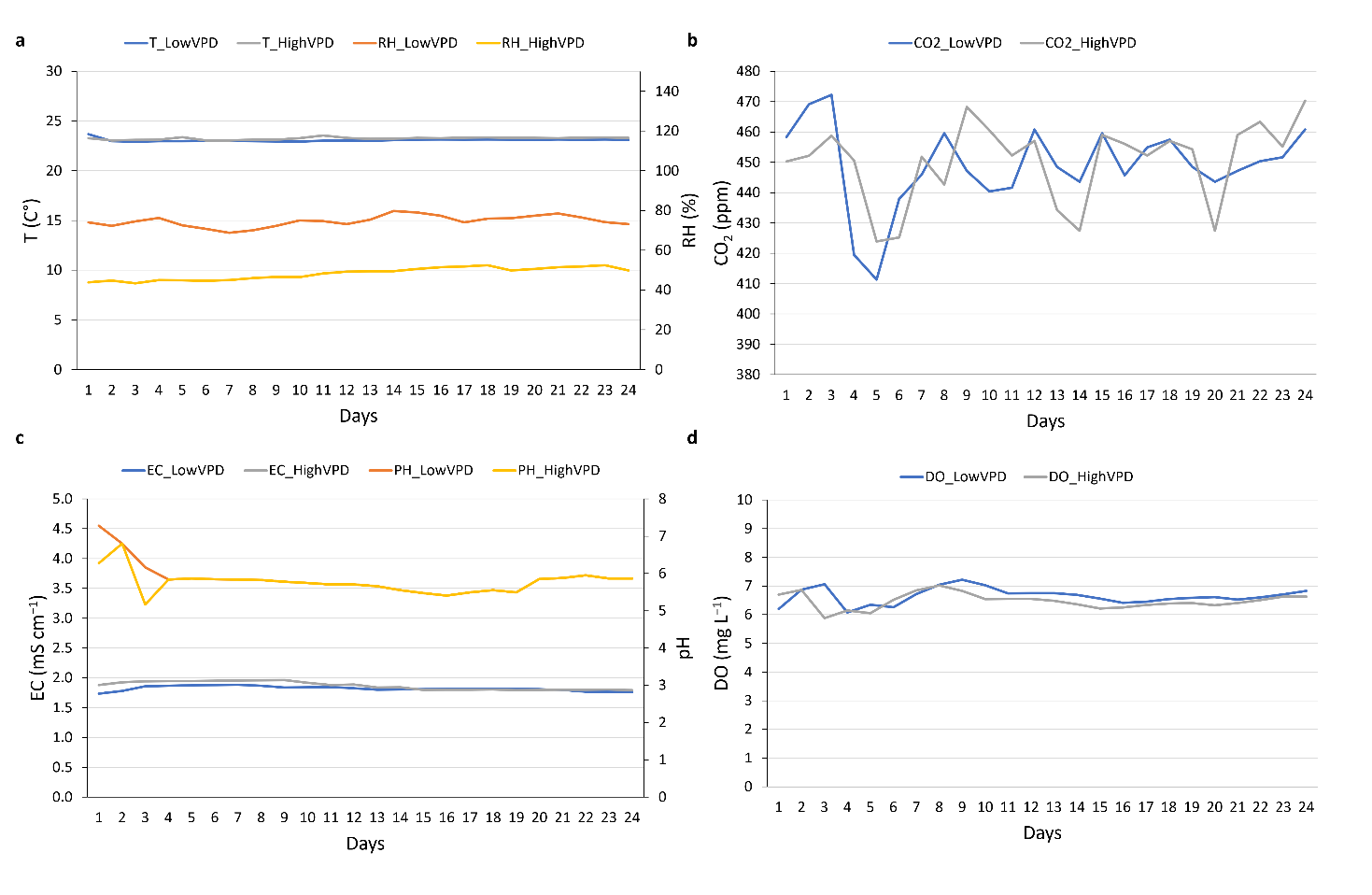


**Fig. S2** Light response curves of lettuces grown under low VPD and high VPD at the three different DLIs (low light, LL; medium light, ML; high light, HL). R^2^ is reported on each curve.


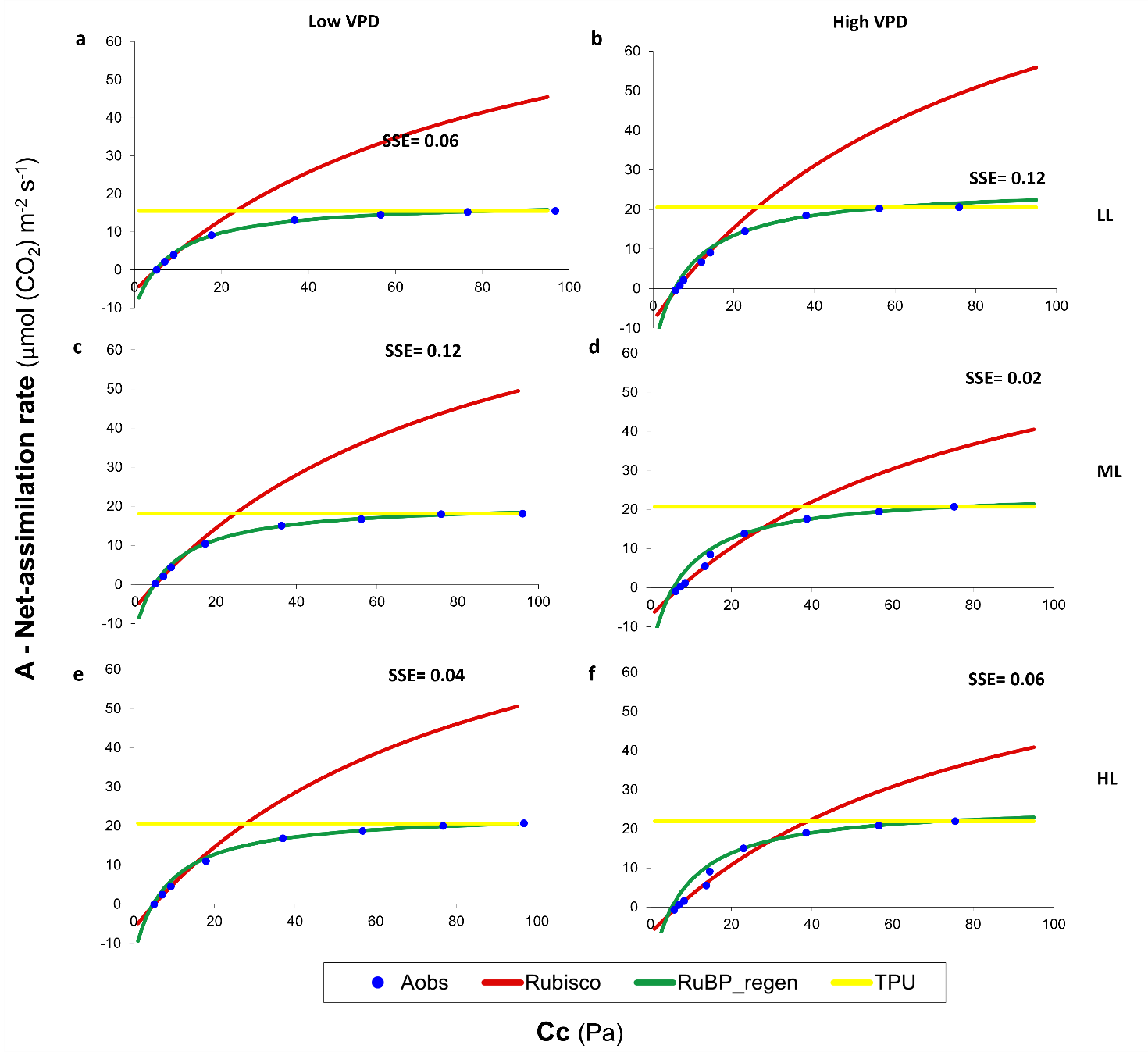


**Fig. S3** CO_2_ response curves of lettuces grown under low VPD and high VPD at the three different DLIs (low light, LL; medium light, ML; high light, HL). R^2^ is reported on each curves.


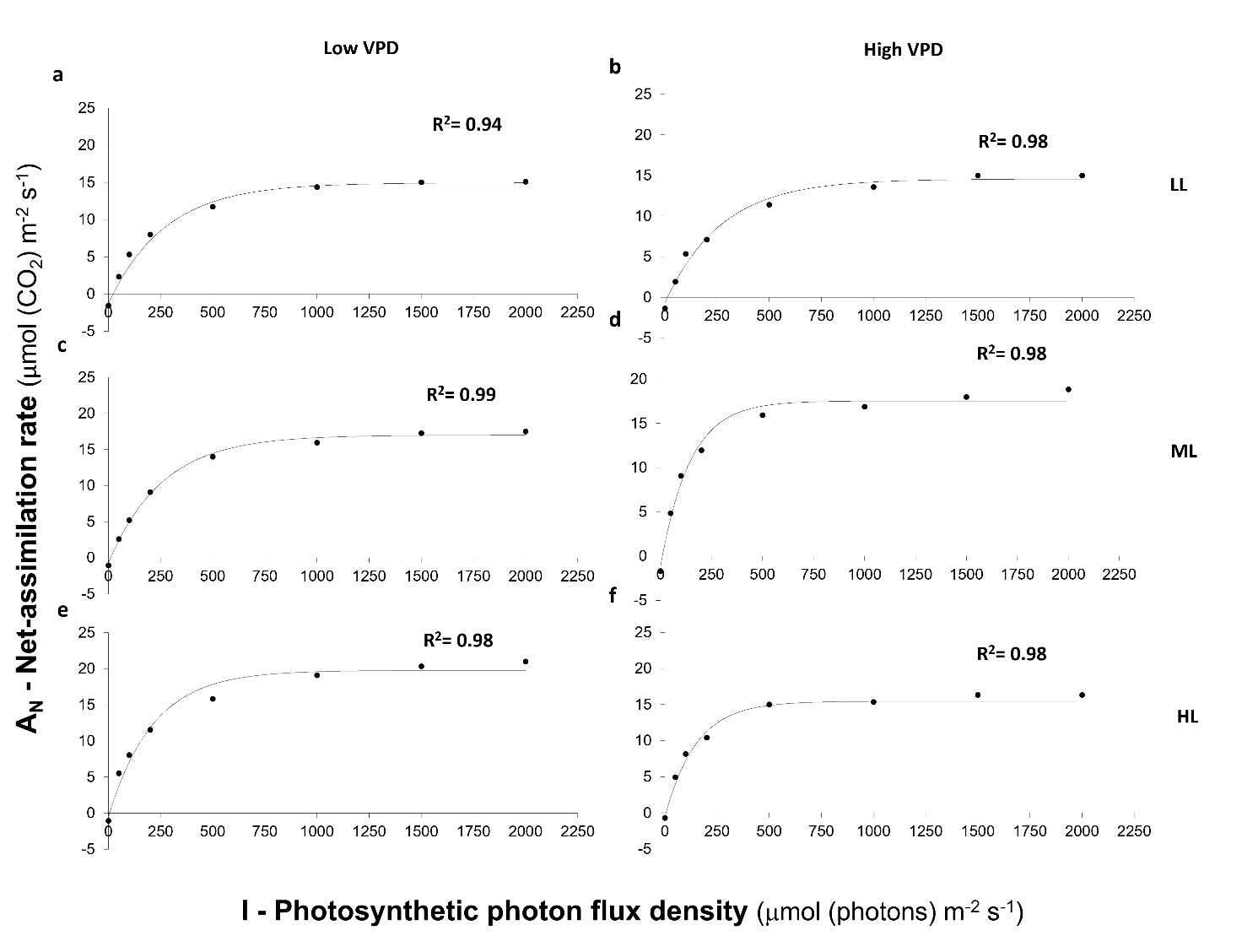

Supplement: Supplementary file 1 — Supplementary file1 (DOCX 722 KB) [file 425_2025_4774_MOESM1_ESM.docx]
